# Foreword to the focus issue: new methodology for developing innovative materials

**DOI:** 10.1080/14686996.2024.2443318

**Published:** 2025-01-31

**Authors:** Hideo Hosono

**Affiliations:** a Institute of Science Tokyo, Yokohama, Japan; b National Institute for Materials Science, Tsukuba, Japan

Research methodology for innovative materials is drastically changing driven by rapidly developing data science and infrastructure for the first-principles calculations on complex condensed matters. The current landscape of materials research totally differs from that of a decade ago.

Japan Science and Technology (JST), a major funder for advanced science and technology under the MEXT, set up the current project within the framework of the Core Research for Evolutional Science and Technology (CREST) Program that facilitates team-type research to produce excellent achievements leading to scientific and technological innovation in 2017. The period of this research project was ~5.5 years, which is the same as other CREST programs. During the period, 4–5 teams were selected each year, resulting in a total of 13 teams picked up from ~100 applications. This focus issue is a collection of the highlighted achievements of each team obtained in this project. Each paper was refereed by 2–3 reviewers.

This research area merges theory, computation, and data science with the experimental sciences, which has been the basis of substance and material development undertaken so far, with the aim of building methods for innovative materials development. Specifically, this research area focuses on the realization of materials or functions that have great societal demand but have not yet been achieved, through new research teaming and methodology, thus aiming at demonstrating the effectiveness of a new integrative approach. This research area covers materials science not only on a simple and clean system but also on complex material system such as sintered polycrystals with many grain boundaries, which is an essential element for the development of practical materials. The research team comprises strong experimental researchers with theoretical/computational, and/or data scientists to promote fusion material research.

This research area cultivates evolutionary and novel methods for materials development to promote industrial competitiveness in our country.

As the leader of the Project and the guest editor of the focus issue, I appreciate each team for submitting the review paper and/or original article to this focus issue. I’m sure this issue exhibits the top-level of current materials research in Japan.

